# Agricultural greenhouses datasets of 2010, 2016, and 2022 in China

**DOI:** 10.1038/s41597-025-05412-y

**Published:** 2025-07-01

**Authors:** Yan Sun, Yuyun Zhang, Jian Hao, Jiang Li, Hengjun Ge, Feifei Jiang, Junna Liu, Xueqing Dong, Jiayuan Guo, Zhanbin Luo, Fu Chen

**Affiliations:** Hohai University, School of Public Administration, Nanjing, 210098 China

**Keywords:** Environmental impact, Agroecology, Sustainability

## Abstract

China has built the world’s largest area of agricultural greenhouse to meet the requirements of climate change and dietary structure changes. Accurate and timely access to information on agricultural greenhouse space is crucial for effectively managing and improving the quality of agricultural production. However, high-quality, high-resolution data on Chinese agricultural greenhouses are still lacking due to difficulties in identification and an insufficient number of representative training data. This study aimed to propose a method for identifying agricultural greenhouse spectral and texture information based on key growth stages using the Google Earth Engine (GEE) cloud platform, Landsat 7 remote sensing images, and combined field surveys and visual interpretation to collect a large number of samples. This method used a random forest classifier to extract spatial information from remote sensing data to create classification datasets of Chinese agricultural greenhouses in 2010, 2016, and 2022. The overall accuracy reached 97%, with a kappa coefficient of 0.82. This dataset may help researchers and decision-makers further develop research and management in facility agriculture.

## Background & Summary

China’s population was 1.408 billion in 2024. The area of agricultural greenhouses in China has continuously increased in the last 20 years to meet the demand for fruits, vegetables, and other products. The Chinese agricultural greenhouses have significantly contributed to enriching agricultural product supply, increasing farmers’ income, and promoting the adjustment of agricultural industry structure^1^. On June 9, 2023, the Ministry of Agriculture and Rural Affairs and three other ministries jointly issued the “National Modern Facility Agriculture Construction Plan (2023–2030)”. The plan aims to expand facility agriculture, targeting 40% of vegetable output and 60% of aquaculture output from facilities by 2030. However, agricultural greenhouses were misused for the large-scale construction of “agricultural garden-style residential areas” in some regions. Then some arable lands were damaged, or even the permanent basic farmlands were illegally occupied. Therefore, timely and accurate acquisition of information on Chinese agricultural greenhouses can provide scientific data support for the government, research institutions, and so forth, and also promote agricultural modernization and sustainable development.

Currently, agricultural greenhouse data are mainly acquired through traditional methods such as sampling surveys and statistical reporting. These methods involve complex workflows, long survey cycles, and excessive human intervention, leading to strong subjectivity in data acquisition, low accuracy, and lack of timeliness^2^. As the remote sensing data with broad coverage and strong timeliness being more easily accessible, it can provide accurate and effective spatial distribution map, and quantify the number of ground agricultural greenhouses^3^. Depending on different research purposes, low-, medium-, and high-resolution images of the remote sensing have various application scenarios. The medium- and low-resolution images contain more bands and spectral information, wider coverage, and longer time series, and are more suitable for large-scale extraction and sequential change monitoring of agricultural greenhouses, compared with high-resolution images. Moderate Resolution Imaging Spectroradiometer (MODIS) dataset, Landsat series, Sentinel series, and so forth^4–6^ are a few commonly used medium- and low-resolution sensor satellites. Aguilar *et al*.^7^ and Li *et al*.^8^ extracted facility planting areas in Almería and distribution information on plastic greenhouses in Xuzhou City based on Landsat 8 images. Novelli^9^ and Sun^10^ extracted distribution information on plastic greenhouses in Almería and Shandong Province based on Sentinel-2 images. When using medium- and low-resolution images for feature extraction, the phenomenon of “same spectrum different objects” and “same object different spectrum” may lead to errors and omissions in the extraction results, influencing classification accuracy. In contrast, high-resolution remote sensing images can capture more detailed target and boundary information, due to their rich details of ground features. When it was used for extracting the agricultural greenhouses, it can increase the classification accuracy, such as high-resolution image series, resource image series, and so forth. For example, based on remote sensing images of HuanJing-1 satellite, the greenhouse vegetable fields in Shouguang City were visually interpreted^11^. Wu *et al*.^12^ have also verified the applicability of different texture extraction algorithms for identifying plastic greenhouses using GaoFen-2 images. Similarly, Zhao *et al*.^13^ and Gao *et al*.^14^ have extracted information of plastic greenhouses in Guantao County (Hebei Province) and Wangyefu Town (Kalaxinqi, Chifeng City, Inner Mongolia) with GaoFen-2 images. However, high-resolution images often have the disadvantages of fewer bands and low spectral resolution, which may increase heterogeneity within the same object and affect land cover identification. Therefore, integrating spectral features, remote sensing indices, and machine learning algorithms to achieve automated extraction of long-term, large-scale, and high-precision agricultural greenhouses may be of great significance.

Machine learning algorithms and remote sensing index methods are mainly used to automatically extract remote sensing information in agricultural greenhouses. Common machine learning algorithms include artificial neural network (ANN)^15,16^, support vector machine (SVM)^17^, classification and regression tree^18^, maximum likelihood classification^19^, nearest neighbor^20^, and random forest classifier^21^. Chen *et al*.^22^ used deep learning to extract agricultural plastic greenhouses in Shouguang, Shandong. They achieved a mean intersection over union (mIOU) of 97.20%. Based on the GEE platform, the random forest classification algorithm was used to produce an agricultural greenhouse dataset (covering 1989–2018) for Shandong, China^23^. Furthermore, the User’s Accuracy and Producer’s Accuracy reached 96.56% and 86.64%, respectively. Remote sensing indices typically include normalized difference vegetation index (NDVI), normalized difference built-up index (NDBI), normalized difference bare soil index, modified normalized difference water index (MNDWI), ratio vegetation index, modified soil-adjusted vegetation index, enhanced water index, and ratio impervious surface index. Remote sensing indices play a crucial role in extracting spatial distribution information of greenhouses by identifying the most suitable feature indices, combined with greenhouse texture features and classification algorithms^24,25^. Yang *et al*.^26^ proposed a new greenhouse index based on spectral, sensitivity, and separability analysis of plastic greenhouses. They used it to extract greenhouses in Weifang District, Shandong, China. The method achieved a kappa coefficient of 0.83 and an overall accuracy of 91.2%. Shi *et al*.^27^ developed a three-step procedure to identify plastic greenhouses in Yucheng, Dezhou, Shandong Province. The identification accuracy reached 95.20%. As a supervised learning method, machine learning often requires manually designed or predefined features. Typically, basic spectral bands are used as features. However, if only raw bands are used, classification accuracy is significantly limited and tends to be low^17^. Therefore, combining remote sensing indices with machine learning algorithms to analyze the spatiotemporal distribution of agricultural greenhouses has certain advantages. In existing studies, publicly available datasets on agricultural greenhouses are scarce. Their spatial coverage is limited, making it difficult to support large-scale and long-term research.

To address this gap, this study aims to create a publicly available dataset of agricultural greenhouses covering multiple time periods and large areas. The agricultural greenhouses annually across mainland China from 2010 to 2022 were mapped. The dataset provides the first complete description of annual agricultural greenhouse dynamics in China over 12 years. It has the potential to support greenhouse-related technologies and guide scientific agricultural planning. And this dataset could support greenhouse pattern analysis, model training, offering a valuable reference for research and policy in agriculture and related fields.

## Methods

### Research framework

**Fig. 1:**
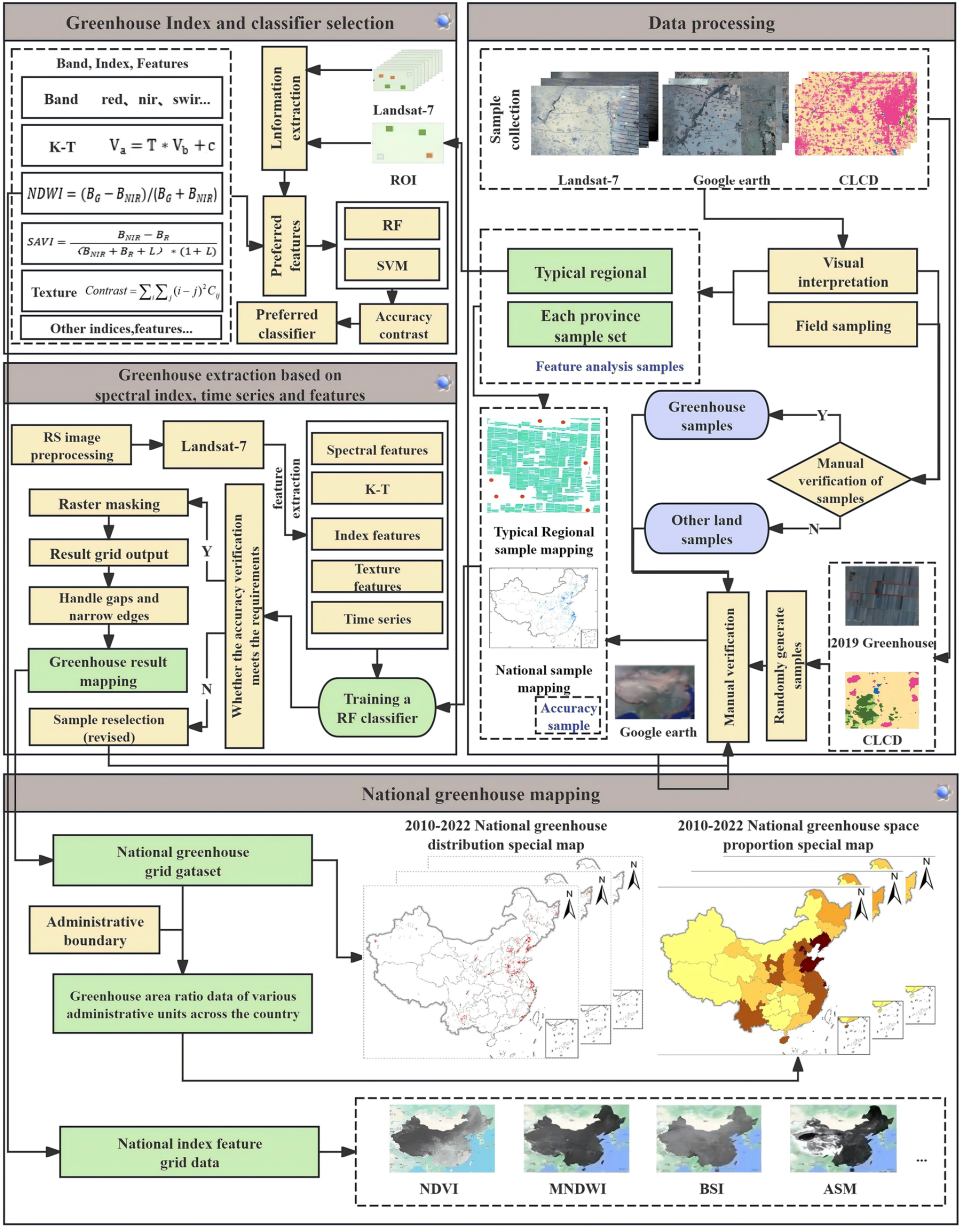
Overall technical roadmap.

The workflow of this study included image preprocessing, sample collection, feature extraction, model training and construction, agricultural greenhouse classification, result postprocessing, accuracy verification, and statistical analysis. First, Landsat 7 image data of China were obtained on the GEE cloud platform, and preprocessing, including cloud interference removal, image stitching, color, and contrast adjustment, was performed to ensure the quality and usability of the images. Second, agricultural greenhouse sample points were collected through visual interpretation and field sampling methods, training and test sets were randomly divided, and a multidimensional feature space was constructed through feature extraction. Finally, a random forest classification model was built to extract national agricultural greenhouse classification data, and postprocessing and accuracy verification were conducted to ensure high-quality national agricultural greenhouse data output. The technical route of this study is depicted in Fig. 1.

### Study area and data source

China is situated on the west coast of the Pacific Ocean, covering a land area of approximately 9.6 million square kilometers, with diverse land types. The terrain of China is distributed in a step-like manner, with higher elevations in the west and lower elevations in the east. The climate of China is complex and diverse, spanning three climate zones: tropical, temperate, and frigid. China has developed a diversified agricultural production system due to varied terrain, landforms, and climate conditions. Agricultural greenhouses are key in protecting crop growth and improving field production efficiency in modern agriculture. They are widely distributed in various provinces in the Chinese mainland, with particularly large numbers in Jiangsu, Shandong, Henan, Hebei, and Liaoning provinces. The continuous innovation and promotion of greenhouse technology have resulted in a steady growth in China’s greenhouses over the past 20 years, far exceeding other countries. However, the spectral characteristics of agricultural greenhouses in remote sensing images vary in different regions and seasons due to geographical location, climatic conditions, and types of crops grown; even the same greenhouse in the same region can have significantly different spectral characteristics in different seasons. These variations pose technical difficulties in accurately identifying and extracting information about agricultural greenhouses based on remote sensing images. Therefore, in-depth research on efficient methods to map agricultural greenhouses in the Chinese mainland is not only of great theoretical significance but also of high practical value in applications.

The data used in this study mainly included remote sensing image collection and land use cover dataset for classification. The data sources and detailed information are presented in Table 1. This study was conducted on the GEE cloud platform. Landsat 7 imagery from 2010, 2016, and 2022 was selected, with a spatial resolution of 30 meters. After preprocessing steps such as cloud removal and mosaicking, we used the data to calculate spectral indices, texture features, and phenological characteristics.

**Table 1:** Data source and description.

Serial number	Data name	Data source	Data format	Data composition	Data purpose
NISL	National-scale Image Sets of Landsat 7 in 2010, 2016, and 2022	Google Earth Engine	Raster	Annual image collections	Large-scale mapping
CLCD	China Land Cover Dataset^28^	Wuhan University	Raster	Global 30-m land cover products from 2000–2022	Sample point collection
NAGP	National Agricultural Greenhouse Product in 2019^29^	Chinese Science Data	Raster	Classification results of the RF algorithm	Classification results

CLCD, China Land Cover Dataset; NAGP, National Agricultural Greenhouse Product in 2019; NISL, National-scale Image Sets of Landsat 7 in 2010; RF, random forest.

In addition, several auxiliary datasets were used for analysis: (1) Land cover data came from the China Land Cover Dataset (CLCD) by Yang *et al*.^28^. This is a nationwide annual classification product at 30-meter resolution. It supports sample interpretation and land cover validation (10.5281/zenodo.4417810). (2) The National Agricultural Greenhouse Product (NAGP) in 2019, published by Feng *et al*.^29^, was used for cross-comparison of the classification results (10.11922/sciencedb.j00001.00230).

### Selection of satellite images and reference samples

The Landsat 7 satellite was launched on April 15, 1999, and began continuous monitoring of the Earth’s surface. It has a revisit cycle of approximately every 14 days and provides global coverage of 30-m resolution image data. It is widely used in various fields, such as land use type identification, forest cover change detection, urban expansion monitoring, and agricultural yield estimation, due to its high accuracy and continuous time series^30^. This study used the GEE platform, selected Landsat 7 optical remote sensing images that had undergone radiometric and atmospheric correction as the basic data source, and preprocessed them by removing clouds and clipping. Images with more than 20% cloud cover were excluded from this study^8,19^. The remaining cloud-free images from different years were synthesized into one image using the median value. Finally, monthly remote sensing images from January to December were synthesized for each year in China, and a high-quality index and feature dataset covering the entire country was constructed, which included monthly averages, maximum and minimum values, phenological characteristics, texture features, and time series features. The dataset was constructed to provide solid data support for the automatic extraction of agricultural greenhouses, so as to achieve rapid and accurate identification and monitoring of agricultural greenhouse distribution. From 2010 to 2022, facility agriculture in China shifted from rapid expansion to stock optimization. In 2010, the policy *Notice on Improving the Management of Facility Agricultural Land* officially classified greenhouses as agricultural production facilities. This removed the earlier requirement for construction land approval, lowered land use barriers, and directly promoted the expansion of greenhouses nationwide. In 2016, No. 1 Document of the Central Committee of the Communist Party of China emphasized including facility agriculture in poverty alleviation projects. It encouraged greenhouse development in poor areas. The *13th Five-Year Plan for Relocated Poverty Alleviation* (Development and Reform Document [2016] No. 2022) also supported income generation for relocated households through facility agriculture. These policies led to a rapid increase in greenhouse area in relatively poor regions of mainland China. In 2022, No. 1 Document of the Central Committee of the Communist Party of China made the regulation of “non-grain land use” a national priority. Large-scale demolition of illegal greenhouses began across the country, and the growth of greenhouse area slowed sharply. To illustrate the development process of greenhouses and considering the sampling years, this study planned to produce annual national greenhouse grid data from 2010 to 2022.

Collecting agricultural greenhouse samples involved a method that combined on-site sampling with visual interpretation. First, several samples were obtained through on-site sampling. Second, high-resolution image data provided by Google Earth and 30-m resolution land use data were used for visual interpretation to further collect agricultural greenhouse samples from various provinces, aiming to increase the density of sample points. During the sampling process, this study followed the principle of “better to lack than to be excessive,” trying to maintain an even distribution of sample numbers in each province, while ensuring the geographical balance of samples to increase the reliability of the samples. Regarding sample distribution, except for provinces with fewer greenhouses, the number of samples in the remaining provinces was not less than 500, with more than half of the provinces having more than 1000 greenhouse samples.

**Fig. 2:**
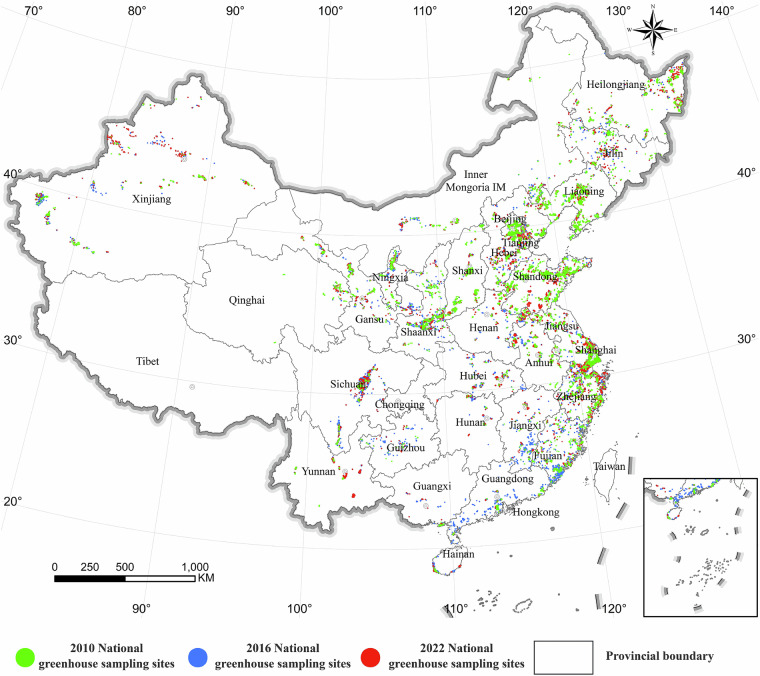
Distribution of national agricultural greenhouse samples from 2010 to 2022.

The sampling time for this study was 2022, with a total of 1,685,431 samples randomly selected. Among these, 135,142 were greenhouse samples and 1,590,335 were non-greenhouse samples (Table 2). Approximately, 6000 samples were obtained through field sampling, and the rest were obtained through visual interpretation. The distribution of samples is depicted in Fig. 2. The selected samples were divided into training and testing sets in a ratio of 4:1, with 1,348,345 samples in the training set and 337,086 samples in the testing set.

**Table 2:** Sample statistics of provincial-level regions in the Chinese mainland.

Year	2010		2016		2022	
Region	Number of greenhouse samples	Number of other feature samples	Number of greenhouse samples	Number of other feature samples	Number of greenhouse samples	Number of other feature samples
Anhui	510	12,774	2,199	22,117	1,339	15,778
Beijing	449	26,275	1,044	10,448	1,004	15,771
Fujian	632	18,541	1,150	18,401	2,689	24,315
Gansu	685	18,479	1,682	19,499	2,302	18,719
Guangdong	204	6,678	547	6,292	1,758	23,769
Guangxi	68	336	329	4,904	522	22,428
Guizhou	139	1,503	264	3,954	740	26,320
Hainan	148	1,434	798	7,188	2,436	18,842
Hebei	2,327	18,501	1,877	19,053	2,053	14,846
Henan	461	10,280	539	7,276	450	14,026
Heilongjiang	841	20,086	2,143	23,541	2,588	22,625
Hubei	180	4,232	1,046	10,043	1,088	16,832
Hunan	64	904	432	6,285	593	17,988
Jilin	669	8,443	2,048	14,023	1,793	19,125
Jiangsu	1,988	20,843	1,356	17,768	1,715	17,312
Jiangxi	152	7,727	443	6,034	1,787	19,573
Liaoning	3,480	24,889	4,274	23,162	5,495	22,882
Inner Mongolia	1,253	19,441	1,207	15,790	1,926	40,678
Ningxia	954	15,168	1,320	13,140	1,934	16,692
Qinghai	108	14,357	885	11,175	1,309	8,912
Shandong	2,028	20,809	3,452	15,590	3,271	12,891
Shanxi	1,407	26,322	1,225	13,879	1,996	23,100
Shaanxi	1,798	24,962	1,672	18,663	3,361	23,997
Shanghai	544	21,764	984	14,640	2,945	18,019
Sichuan	1,045	21,186	2,200	19,100	6,805	42,086
Tianjin	466	24,622	862	8,174	1,199	14,144
Xinjiang	956	22,185	3,606	28,171	5,674	105,535
Yunnan	511	11,562	1,586	16,024	1,913	22,342
Zhejiang	2,261	23,996	2,501	23,243	2,458	24,866
**Total**	**26,328**	**448,299**	**43,671**	**417,577**	**65,143**	**684,413**

### Greenhouse extraction

#### Feature extraction

The automatic identification process of agricultural greenhouses mainly relies on the differences in spectral, temporal, and texture characteristics and other land type features, and accurately extracts them through a series of established rules. However, traditional classification methods only rely on limited spectral features, which makes distinguishing agricultural greenhouses from different land types with high spectral similarity difficult.

Therefore, this study constructed a multidimensional feature space to enhance the discrimination between greenhouses and other land object categories through in-depth analysis and research on different land object indices and characteristics. The key feature extraction indicators selected through comparative analysis included crown transformation features, spectral indices, texture features, and time series features. The features were calculated using the GEE platform.

**Fig. 3:**
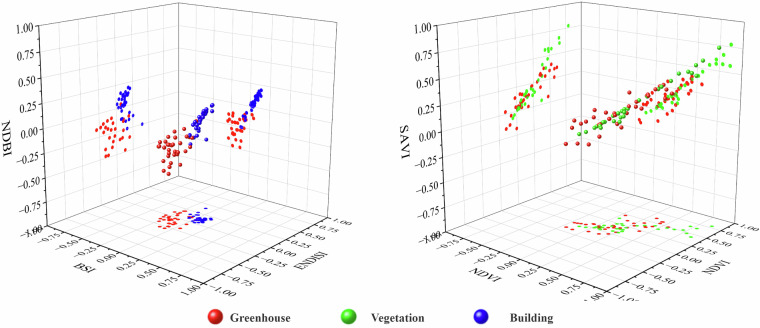
Spatial characteristics of various regional price indices.

**Fig. 4:**
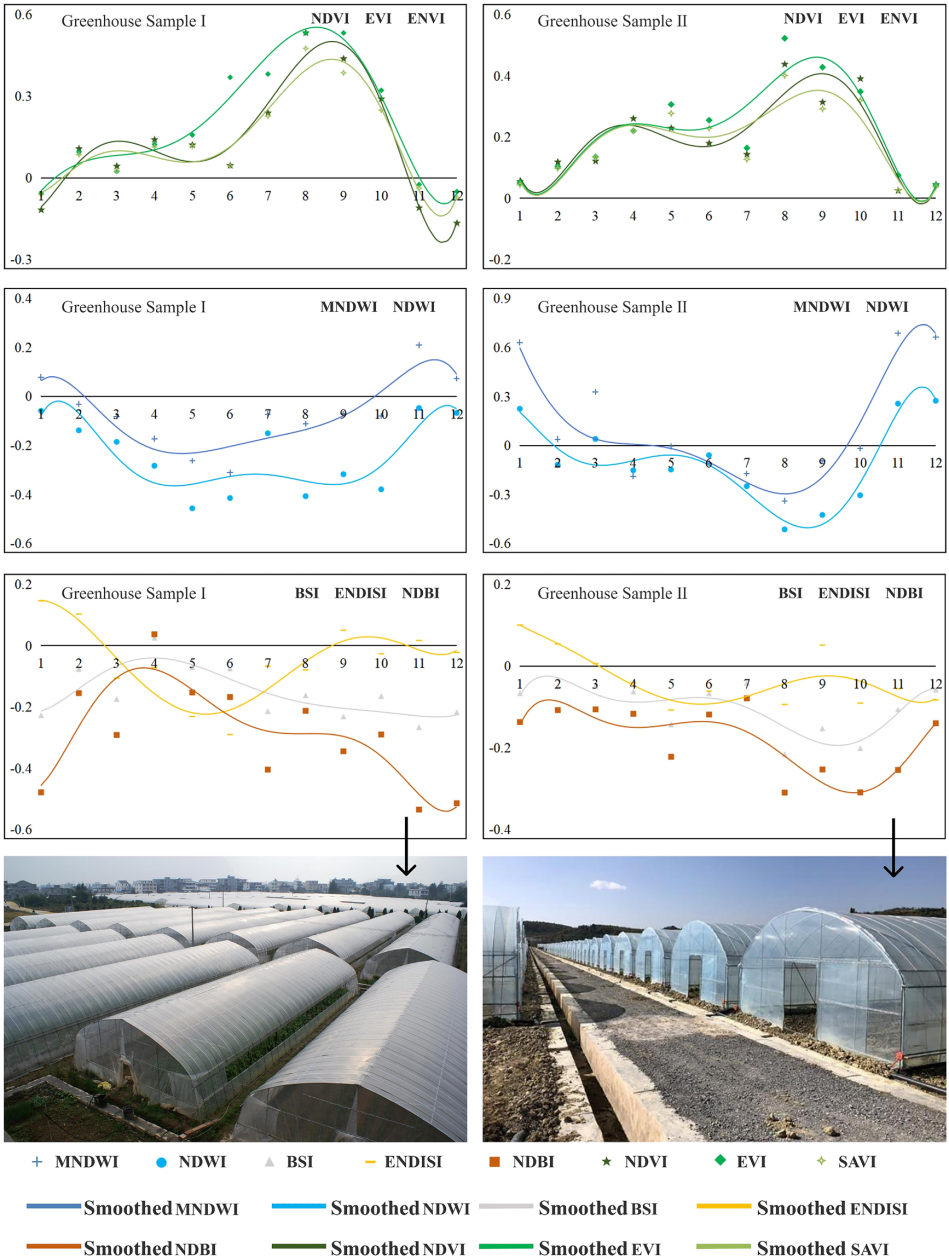
Trend of different indices in greenhouse samples.

**Fig. 5:**
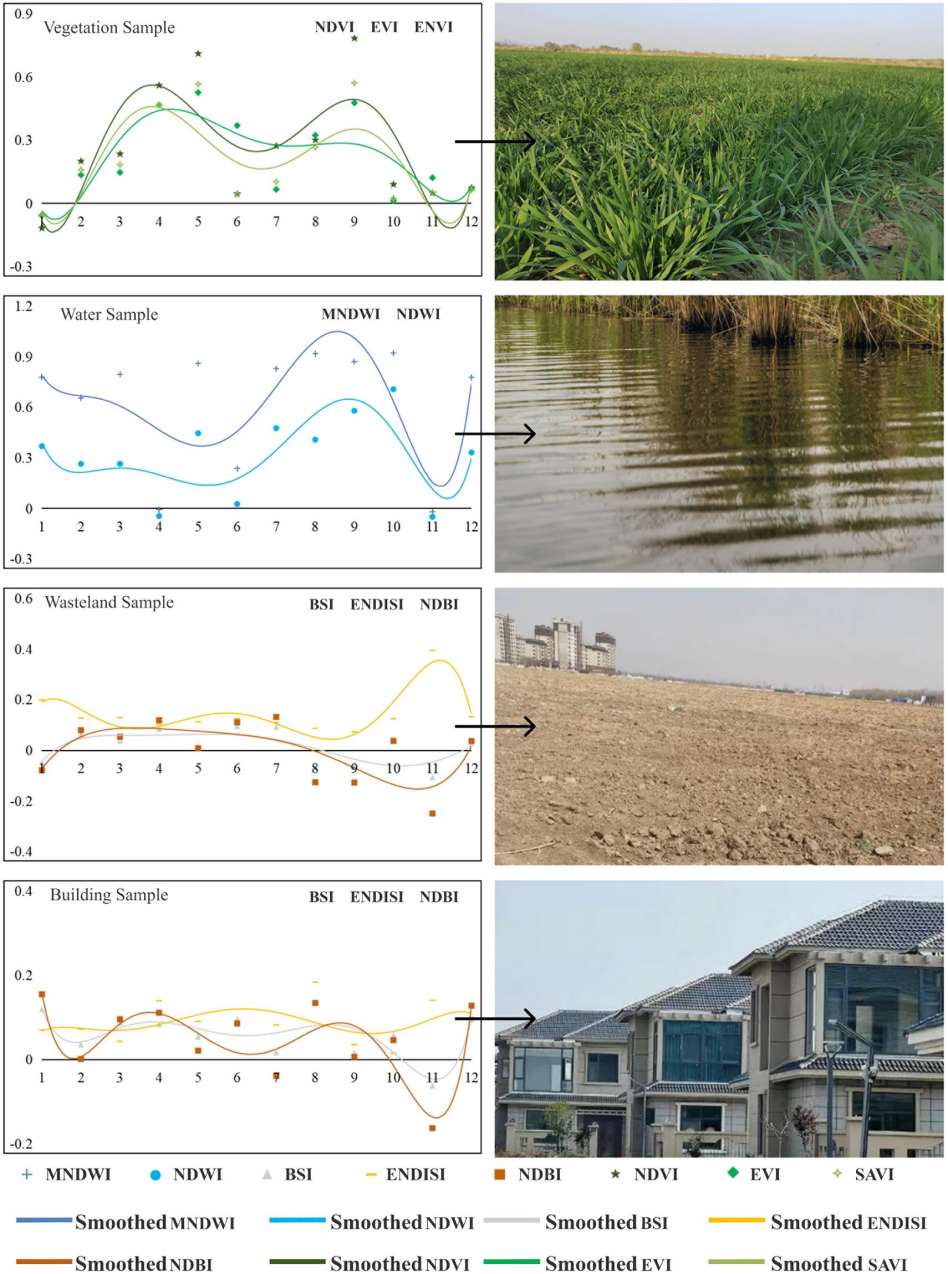
Trend of different indices in other land cover samples.
  Therefore, this study further compared the temporal changes in spectral index features of greenhouse samples with those of other land cover samples based on spectral index feature extraction to improve the accuracy of identifying greenhouses. Temporal spectral index features refer to the monthly values of spectral index features, and the calculation formula for monthly spectral index values was the same as formulas (2–9).

1. Tasseled-cap transformation
  The Tasseled-cap transformation (TCT) is a technique for converting the original bands of an image into a new set of bands more sensitive to vegetation, typically including brightness, greenness, and wetness (soil or surface moisture) as the three main features. This effectively improves the separability between agricultural greenhouses, watered fields, and open vegetation. The formula for TCT is as follows:
  1 $${T\ast V}_{b}+c$$
  where *V*_*a*_ represents the pixel vector of the multispectral space after transformation; *V*_*b*_ represents the pixel vector of the multi-multispectral space before transformation; *c* represents a constant; and *T* represents the transformation matrix.
2. Spectral index
  The spectral index characterizes features such as land cover types, growth status, vegetation content, and soil properties. Therefore, this study used a literature review and experimental comparison to select eight different spectral indices to further improve the separability of agricultural greenhouses based on the application of TCT technology. These indices included normalized difference water index (NDWI), MNDWI, NDBI, NDVI, soil-adjusted vegetation index (SAVI), bare soil index (BSI), enhanced nonphotosynthetic vegetation index (ENDISI), and enhanced vegetation index (EVI). Indices were selected based on their ability to identify and analyze specific features under different environments and conditions. The calculation formulas for spectral indices are as follows:
  2 $${\rm{NDWI}}=\frac{{B}_{{\rm{G}}}-{B}_{{\rm{NIR}}}}{{B}_{{\rm{G}}}+{B}_{{\rm{NIR}}}}$$
  3 $${\rm{MNDWI}}=\frac{{B}_{{\rm{G}}}-{B}_{{\rm{SWIR}}1}}{{B}_{{\rm{G}}}+{B}_{{\rm{SWIR}}1}}$$
  4 $${\rm{NDBI}}=\frac{{B}_{{\rm{SWIR}}1}-{B}_{{\rm{NIR}}}}{{B}_{{\rm{SWIR}}1}+{B}_{{\rm{NIR}}}}$$
  5 $${\rm{NDVI}}=\frac{{B}_{{\rm{NIR}}}-{B}_{{\rm{R}}}}{{B}_{{\rm{NIR}}}+{B}_{{\rm{R}}}}$$
  6 $${\rm{SAVI}}=\frac{{B}_{{\rm{NIR}}}-{B}_{{\rm{R}}}}{{(B}_{{\rm{NIR}}}+{B}_{{\rm{R}}}+L)\ast (1+L)}$$
  7 $${\rm{BSI}}=\frac{{(B}_{{\rm{R}}}-{B}_{{\rm{SWIR}}1})-({B}_{{\rm{NIR}}}+{B}_{{\rm{G}})}}{{(B}_{{\rm{R}}}+{B}_{{\rm{SWIR}}1}+{B}_{{\rm{NIR}}}+{B}_{{\rm{G}}})}$$
  8 $${\rm{ENDISI}}=\frac{{(2\ast B}_{{\rm{B}}}+{B}_{{\rm{SWIR}}2})\ast \frac{1}{2}-{(B}_{{\rm{R}}}+{B}_{{\rm{NIR}}}+{B}_{{\rm{SWIR}}1})\ast \frac{1}{3}}{{(2\ast B}_{{\rm{B}}}+{B}_{{\rm{SWIR}}2})\ast \frac{1}{2}+{(B}_{{\rm{R}}}+{B}_{{\rm{NIR}}}+{B}_{{\rm{SWIR}}1})\ast \frac{1}{3}}$$
  9 $${EVI}=2.5\ast \frac{{B}_{{\rm{NIR}}}-{B}_{{\rm{R}}}}{{B}_{{\rm{NIR}}}+6\ast {B}_{{\rm{R}}}-7\ast {B}_{{\rm{B}}}+1}$$
  where, *B*_G_, *B*_R_, *B*_NIR_, *B*_SWIR1_ and *B*_SWIR2_ represent the reflectance values of the blue band, green band, red band, near-infrared band, shortwave infrared band 1, and shortwave infrared band 2, respectively. These band reflectance values correspond to bands 1, 2, 3, 4, 5 and 7 in Landsat 7 satellite images. *L* represents a correction factor used to adjust and optimize the calculation process. After multiple experiments and tests, the correction factor *L* was determined to be 0.2.
3. Texture feature extraction
  Visual interpretation analysis of high-resolution image data shows that agricultural greenhouses exhibit significant characteristics in terms of geometric shape and surface texture compared with other land features. Agricultural greenhouses are usually distributed in groups, and different groups exhibit different characteristics. Specifically, sunlight greenhouses comprise gables, back walls, support frames, and covering materials, mostly appearing black or gray-green, and usually maintain a distance of about 2 m between greenhouses. The spacing between connected greenhouses is relatively small, with a longer overall length, predominantly exhibiting a silver-white appearance. Plastic greenhouses mainly use plastic film as covering materials, with relatively shorter individual lengths and more obvious spacing between them, mostly appearing white or gray-white.
  This study selected six most representative texture features, including angular second moment, contrast (CON), correlation (COR), entropy (ENT), variance (VAR), and dissimilarity (DIS), to more accurately distinguish differences between greenhouses and other land covers. Four commonly used moving window sizes were adopted, namely, 3 × 3, 5 × 5, 7 × 7, and 11 × 11, to compare the effects of different window sizes on texture features. After a series of comparisons and analyses, each texture feature produced the highest classification accuracy in the 3 × 3 moving window. Therefore, the moving window size for texture feature analysis was determined to be 3 × 3. The calculation formulas for texture features are as follows:
  10 $${ASM}=\mathop{\sum }\limits_{i,j=0}^{n-1}{{P}_{i,j}}^{2}$$
  11 $${CON}=\mathop{\sum }\limits_{i,j=0}^{n-1}{P}_{i,j}{(i-j)}^{2}$$
  12 $${COR}=\mathop{\sum }\limits_{i,j=0}^{n-1}{P}_{i,j}[\frac{(i-{\mu }_{i})\ast (j-{\mu }_{j})}{{\sigma }_{i}\ast {\sigma }_{j}}]$$
  13 $${ENT}=\mathop{\sum }\limits_{i,j=0}^{n-1}{P}_{i,j}(\,-\mathrm{ln}\,{P}_{i,j})$$
  14 $${VAR}=-\,\mathop{\sum }\limits_{i,j=0}^{n-1}{P}_{i,j}{(i-\frac{{P}_{i,j}}{2})}^{2}$$
  15 $${DIS}=\mathop{\sum }\limits_{i,j=0}^{n-1}{P}_{i,j}|i-j|$$
  where *n* represents the gray level, and *P*_*i,j*_ is the normalized gray value of the co-occurrence matrix element.
4. Temporal characteristics
  The representation and attributes of different land objects show diverse changes in different seasons^31^. These changes directly impact the spectral characteristics of land objects, which, in turn, affect the analysis and interpretation of remote sensing images. Generally, various spectral index values exhibit significant heterogeneity due to seasonal precipitation patterns, temperature changes, vegetation changes, and human activities. For example, the NDVI, NDWI, and MNDWI values are often higher in spring and summer when vegetation growth is vigorous and precipitation is relatively high. In contrast, these values are lower in autumn and winter when vegetation withers or goes dormant and precipitation is relatively low. In addition, NDBI values are usually higher in densely built areas, and when vegetation cover is low, BSI is more suitable for distinguishing between bare soil and built-up land. This study compared the spatial differences in the mean values of typical land object spectral indices to more clearly reveal the temporal differences in various spectral indices and presented their time series change statistics (Figs. 3–5). For most of the year, the NDWI and MNDWI values of water bodies were greater than 0, the NDBI and BSI indices of buildings were greater than 0, whereas the corresponding values of greenhouses were less than 0. In terms of vegetation indices, although the corresponding values of greenhouses were similar to those of farmland and showed a bimodal trend, the first peak was significantly lower than the second peak, the duration of the valley was shorter than that of the farmland, and the highest peak value was also significantly lower than that of farmland vegetation indices.

#### Classifier selection and operation

Random forest (RF), SVM, and ANN, and other machine learning algorithms have been widely used in remote sensing land cover classification research. RF is an ensemble learning classifier composed of multiple decision tree models, which can effectively reduce model overfitting and improve robustness. RF mainly calculates the importance of features using the Gini index for feature selection, allowing us to select only high-scoring classification feature variables and avoid interference from redundant and irrelevant features. SVM is a machine learning algorithm based on statistical learning. The SVM maps feature vectors to high-dimensional feature space and determines the hyperplane that best separates different categories for classification. This algorithm has high generalization ability and is particularly suitable for situations with few samples. ANN is built on the basic principles of biological neural networks. After understanding and abstracting the brain structure and external stimulus response mechanism, ANN simulates the processing mechanism of the neural system in the brain for complex information based on network topology knowledge. This model is known for its parallel distributed processing capabilities, high fault tolerance, intelligence, and self-learning characteristics.

**Fig. 6:**
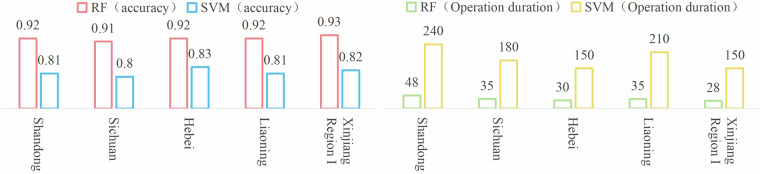
Classification accuracy and performance of different classifiers of representative provinces. Note: Operation duration unit is minute (min).

However, ANNs are more suitable for fine classification of small-scale, high-resolution images and unsuitable for large-scale greenhouse extraction nationwide^32^. Therefore, this study first selected several representative provinces to conduct classification tests using SVMs and RFs, and then recorded the final accuracy and computation time of the test areas. A comparative analysis was conducted, which showed that the RF model did not require data normalization when processing multiple types and dimensions of features, and its accuracy was higher and computation time was significantly lower than those of SVM classification (Fig. 6). Therefore, in the end, this study chose to train and classify based on the RF model aiming to reduce computational complexity and ensure higher classification accuracy.

**Fig. 7:**
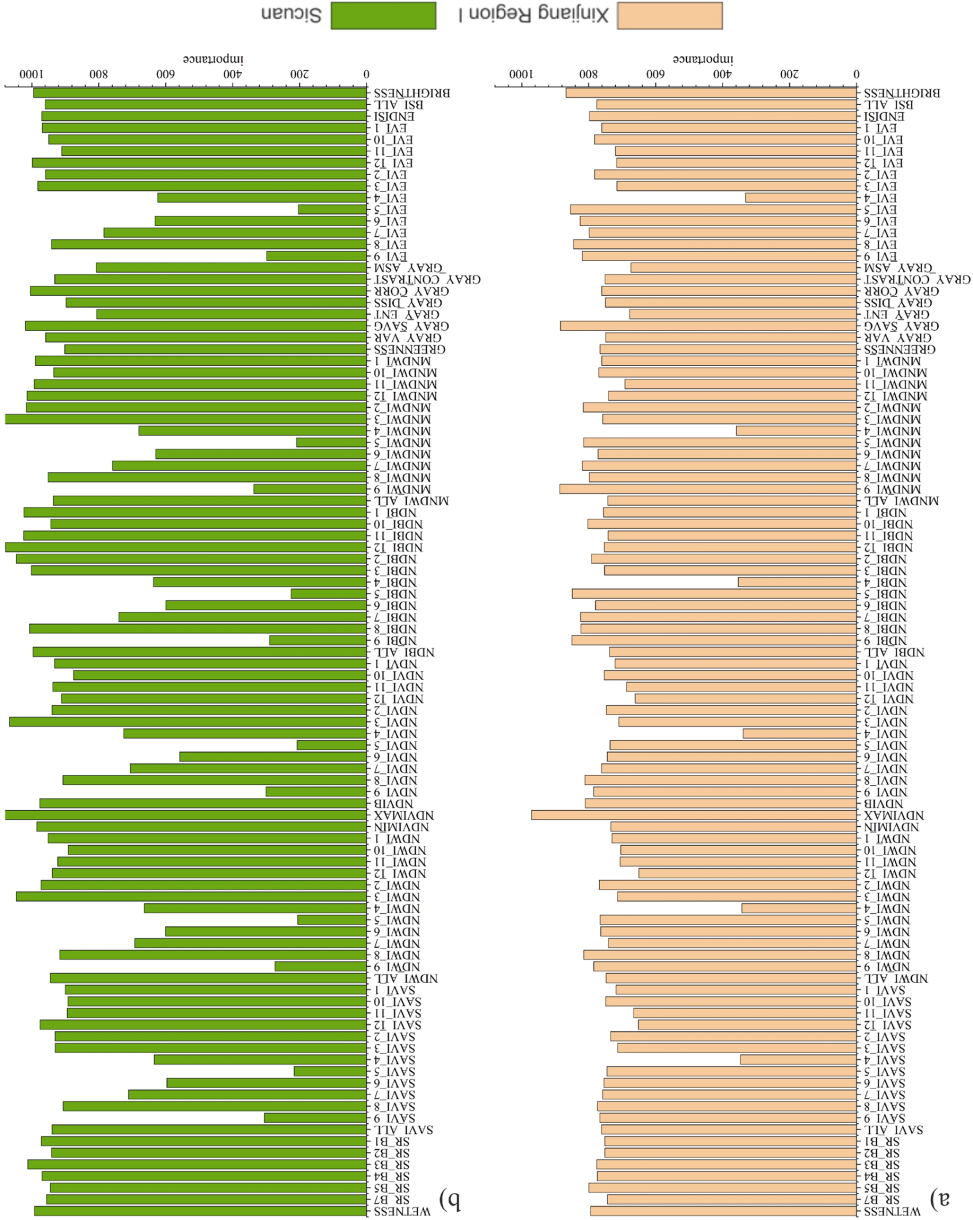
Feature importance of random forest models in typical regions: (**a**) Xinjiang, and (**b**) Sichuan.

**Fig. 8:**
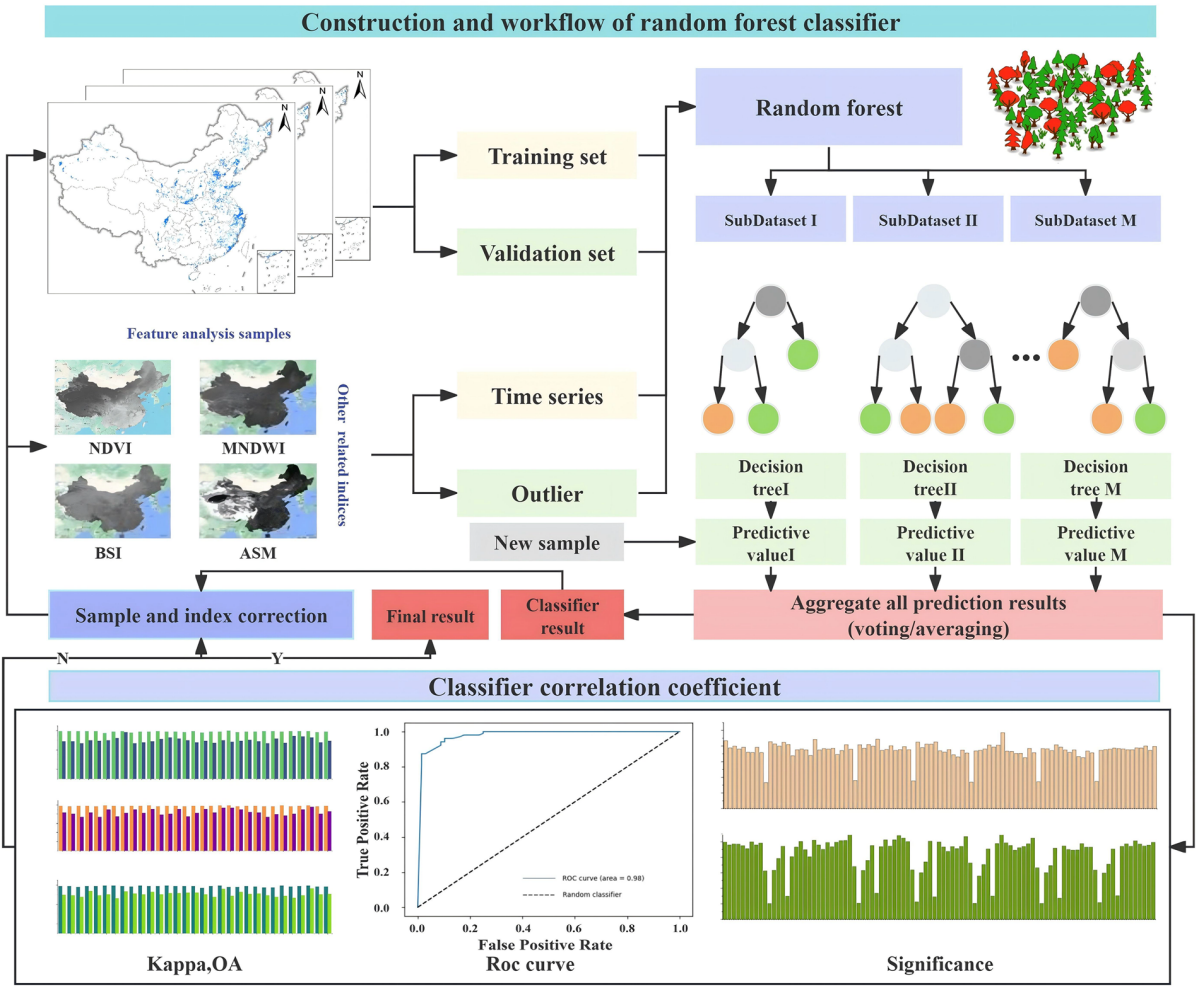
Classifier construction and workflow.

Parameter adjustment is the best method to improve the classification accuracy of machine learning models. The RF model has fewer parameters, with the number of decision trees (ntree) and the number of features randomly selected during the training of each decision tree (mtry)^33^ being the important ones. Generally, increasing ntree effectively reduces the model’s generalization error, but decreases the computational efficiency; mtry determines the classification ability of individual decision trees and also affects the relationship between decision trees. This study used R language programming to test parameter settings. First, the parameter values with the highest accuracy were selected, and then the smallest parameters were selected under the accuracy conditions to optimize efficiency and accuracy^34^. Adding random features improves classification accuracy, but high-dimensional features may have similarities between them, which can interfere with classification ability and reduce computational efficiency. Therefore, selecting feature variables is crucial for the model’s performance. After repeated debugging, when ntree = 200, the model fitting converged and the classification results were optimal. Considering accuracy, statistical error, and computational efficiency, no limit was set on the maximum number of leaf nodes to ensure that the complexity did not impact the classification results. Setting mtry to the square root of the number of input features maintained classification effectiveness while reducing computational load. A normalized importance ranking based on the Gini coefficient can quantitatively measure the contribution of each feature^33,35^, gradually reducing input feature dimensions while ensuring classification performance and efficiency to achieve dimensionality reduction. Figure 7 shows the feature importance of typical regions in this study, with features of low importance removed when constructing the classifier in Fig. 8.

## Data Records

In this study, a dataset containing detailed information was successfully constructed, which included grid data of agricultural greenhouses in various regions of China in 2010, 2016, and 2022. This dataset was named “China GH.” The dataset can be downloaded at 10.6084/m9.figshare.28559747^36^. The constructed China GH dataset includes not only precise geographical coordinates of agricultural greenhouses nationwide but also detailed records of the distribution of sample points in each greenhouse. The China GH dataset is presented in Tag Image File Format (TIFF) and follows the World Geodetic System-1984 Coordinate System (WGS-84). Additionally, the research team is committed to updating the map of Chinese agricultural greenhouses annually to maintain the timeliness and accuracy of the information. These carefully crafted maps of agricultural greenhouses will be publicly released in a timely manner for research and reference use upon completion.

## Technical Validation

### Agricultural greenhouse classification results in the Chinese mainland

#### Overall result

**Fig. 9:**
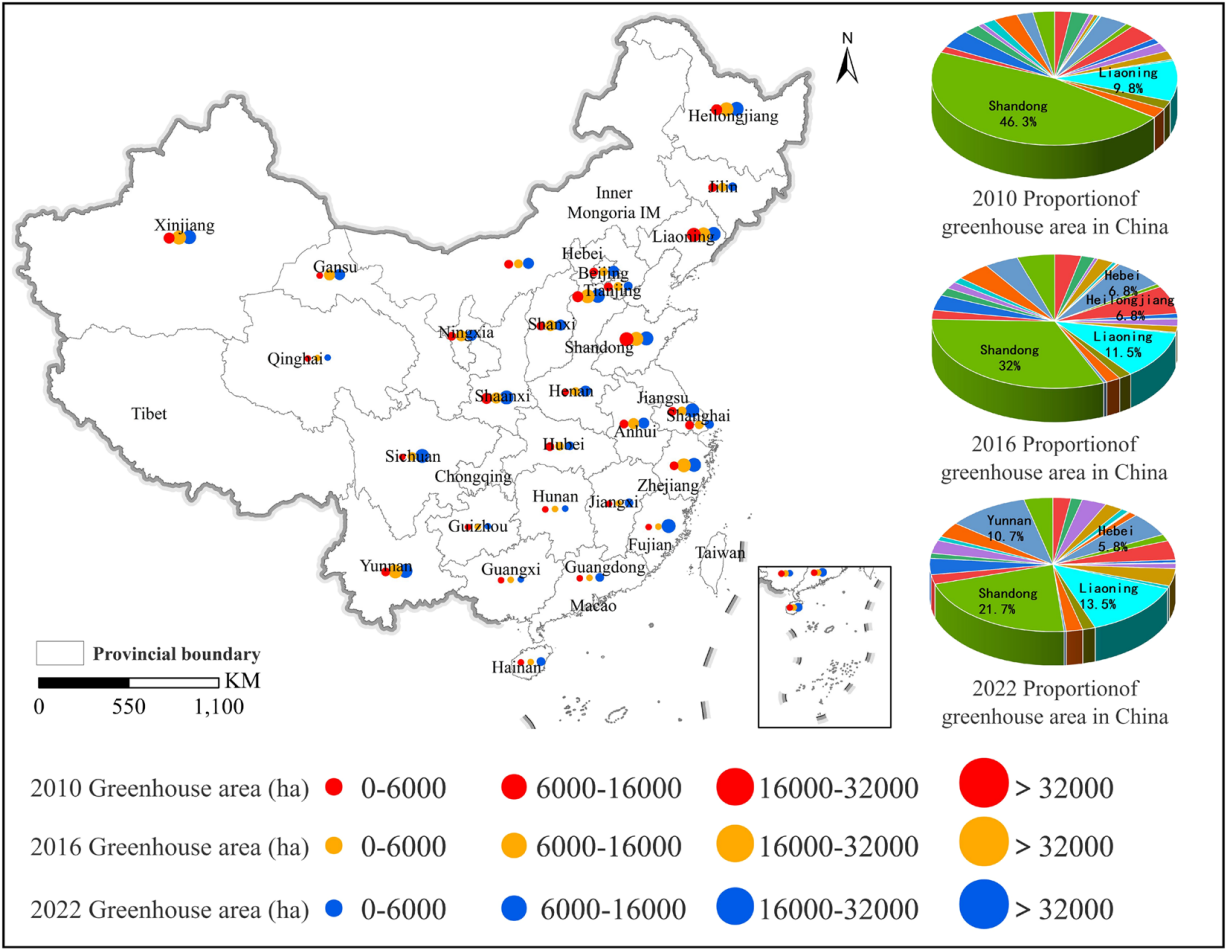
Statistical results of the distribution area of agricultural greenhouses in the Chinese mainland.

**Fig. 10:**
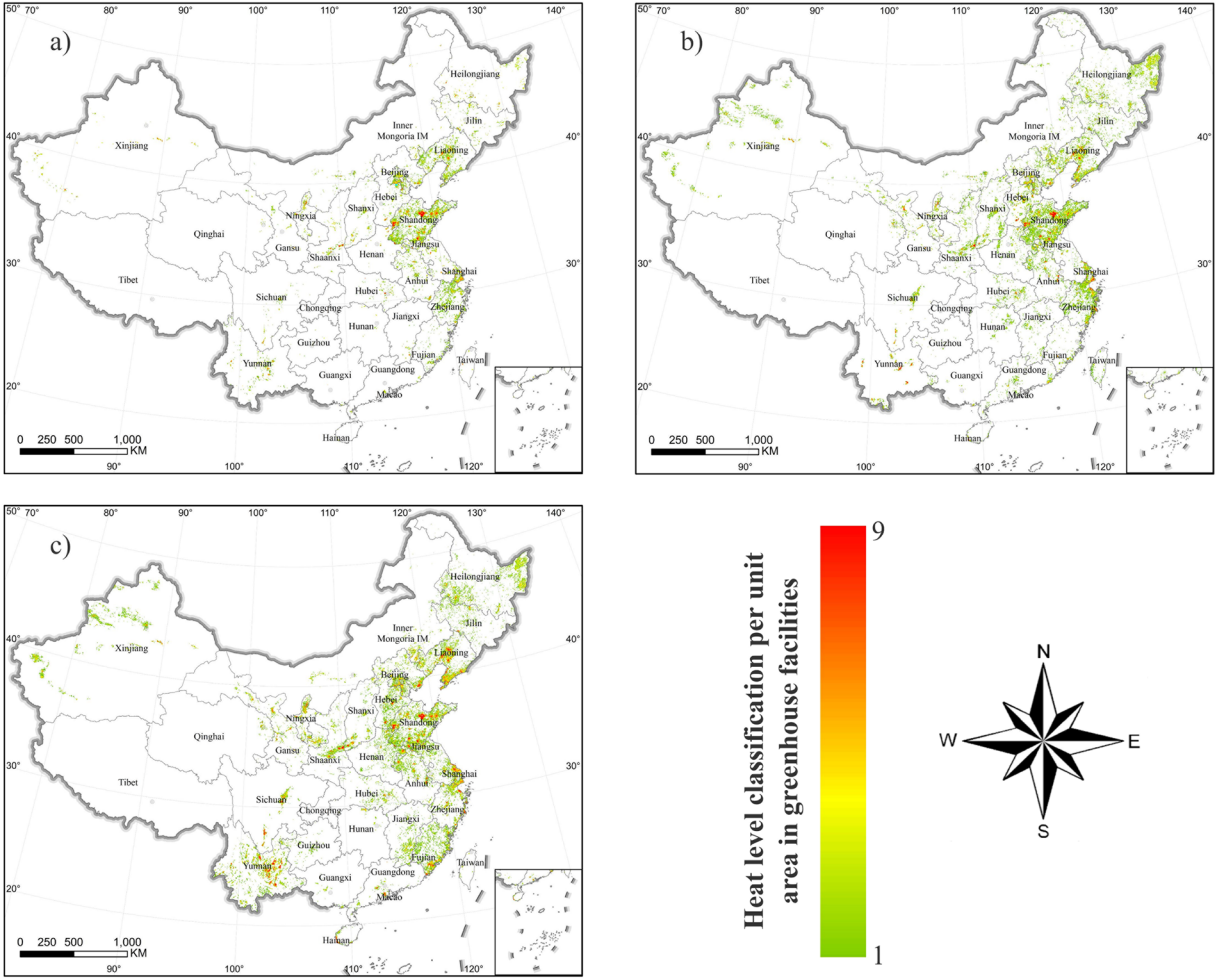
Thermal map of the spatial distribution of agricultural greenhouses in the Chinese mainland: (**a**) 2010; (**b**) 2016; (**c**) 2022.

**Fig. 11:**
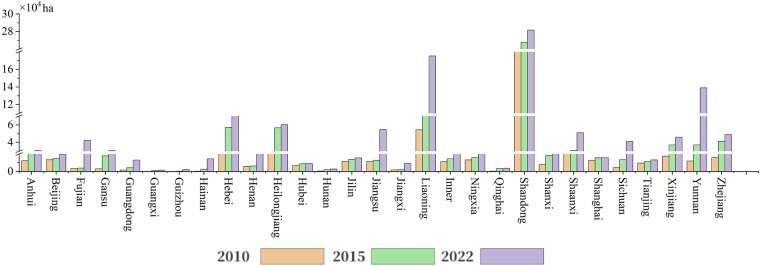
Statistical results of the area of agricultural greenhouses in China.

The planting area of agricultural greenhouses in China showed a significant growth trend from 2010 to 2022 (Fig. 9, Table 3). In 2010, the total planting area of agricultural greenhouses nationwide was 555,460 ha, which increased to 835,923 ha in 2016, and further to 1,295,480 hectares in 2022. Thus, the planting area of agricultural greenhouses nationwide continued to expand at a rate of nearly 50% every 6 years. In 2010, the agricultural greenhouses in China were relatively concentrated in eastern regions such as Shandong, Beijing, and Liaoning; however, by 2022, the area of greenhouses in western regions such as Yunnan and Xinjiang had rapidly increased (Figs. 10, 11).

**Table 3:** Statistics of agricultural greenhouse area in provincial-level regions in Chinese mainland.

Region	Greenhouse area in 2010 (ha)	Greenhouse area in 2016 (ha)	Greenhouse area in 2022 (ha)
Anhui	12,176.34	29,093.87	30,577.28
Beijing	13,308.02	14,839.27	19,371.74
Fujian	3,508.54	3,830.52	42,270.44
Gansu	3,040.43	17,766.29	30,746.24
Guangdong	1,643.73	4,313.00	13,000.70
Guangxi	1,86.29	998.46	1,332.81
Guizhou	247.47	269.48	2,107.63
Hainan	300.63	2,547.47	14,326.32
Hebei	20,717.69	57,018.43	74,808.60
Henan	5,478.61	6,253.36	20,723.60
Heilongjiang	22,432.46	56,657.00	60,194.68
Hubei	6,807.99	8,799.42	9,097.43
Hunan	527.79	2,210.80	2,800.83
Jilin	11,546.10	13,913.70	1,5521.99
Jiangsu	11,628.85	12,686.78	54,696.67
Jiangxi	1,797.94	2,184.52	9,153.63
Liaoning	54,365.89	96,129.84	17,5229.97
Inner Mongolia	11,192.55	14,424.78	21,658.36
Ningxia	13,130.34	16,122.26	28,283.36
Qinghai	399.52	3,511.62	3,592.89
Shandong	257,390.29	267,550.42	28,1734.96
Shanxi	8,135.73	18,340.49	29,711.02
Shaanxi	23,804.12	30,730.12	50,938.69
Shanghai	12,641.19	15,572.57	15,653.31
Sichuan	4,498.87	13,550.15	41,018.50
Tianjin	9,490.99	11,186.33	13,079.44
Xinjiang	17,336.32	37,045.58	45,874.87
Yunnan	11,856.82	37,046.28	139,078.70
Zhejiang	15,867.46	41,329.77	48,895.25
Total	555,458.97	835,922.58	1,295,479.91

#### Greenhouse space information classification statistical results in typical provinces

**Fig. 12:**
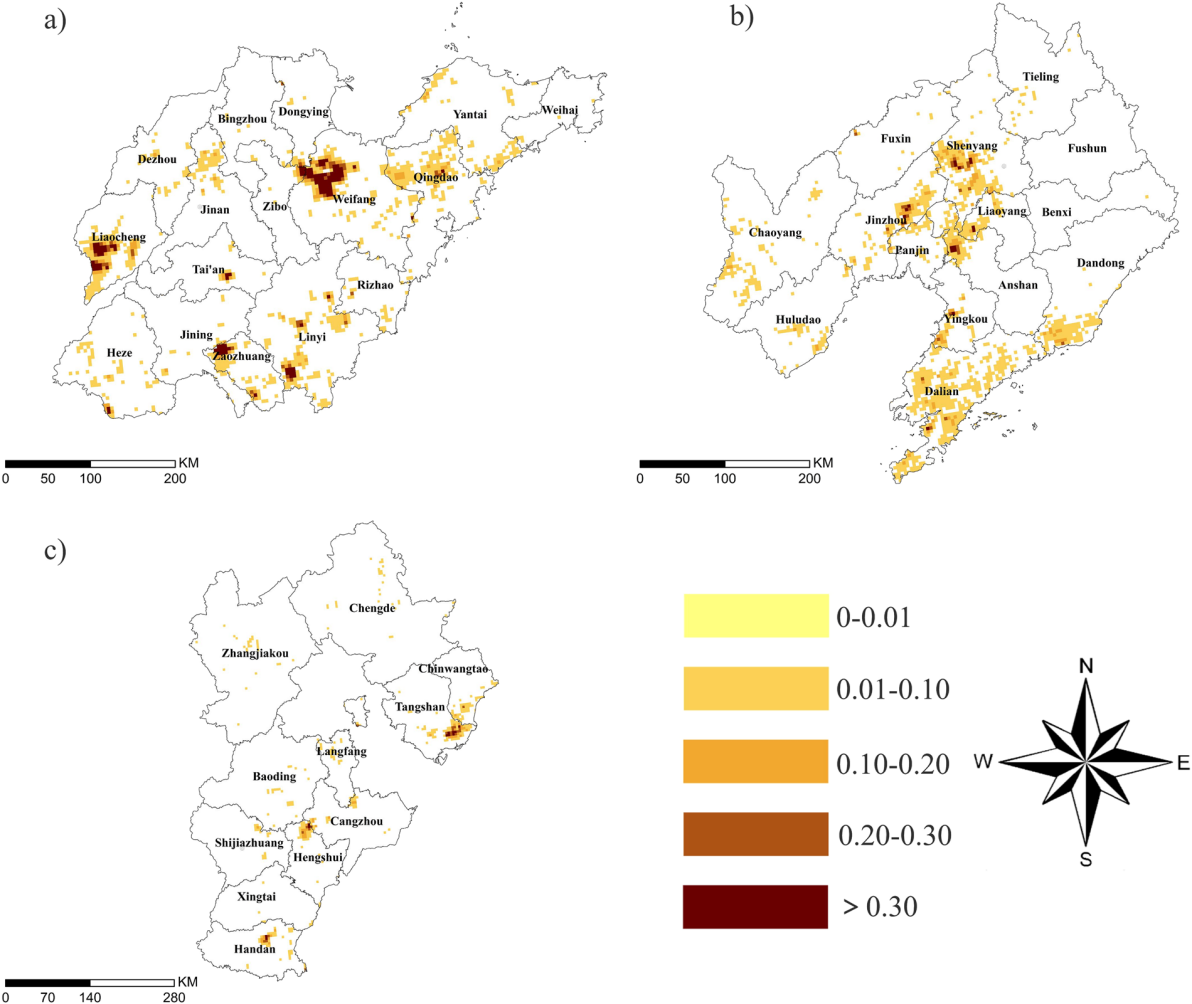
Proportion of greenhouse areas in typical provinces within a 5-km grid: (**a**) Shandong Province; (**b**) Shandong Province; (**c**) Hebei Province.

This study produced the classification results of agricultural greenhouses in typical greenhouse planting areas in China in 2022 (Fig. 12). For example, the distribution of agricultural greenhouses in Shandong Province was mainly concentrated in cities such as Qingzhou, Shouguang, Luling County, and Shen County. The agricultural greenhouse planting areas in Liaoning Province were mainly distributed in Xinmin City, Beizhen City, Haicheng City, and the southern coastal areas. The distribution of agricultural greenhouses in Hebei Province was relatively extensive, with significant greenhouse planting activities in Raoyang County, Leting County, and Yongnian District.

### Evaluation of the accuracy of agricultural greenhouse classification results

#### Evaluation of overall classification accuracy

After classifying the provincial administrative regions, this study evaluated the classification accuracy using a test sample set, calculating accuracy verification indicators such as overall accuracy (OA) and the kappa coefficient. As shown in Table 4, the OA of the classification for each province in 2010, 2016, and 2022 all exceeded 94%, with kappa coefficients all greater than 0.7. A Kappa coefficient between 0.61 and 0.80 is considered “substantial agreement,” while a value between 0.81 and 1.00 is classified as “almost perfect agreement”^37^. A Kappa coefficient above 0.7 indicates a high reliability of the classification results. It is worth noting that with the rapid expansion of facility agriculture, the spatial distribution of greenhouses has changed significantly. Greenhouse types have become more diverse, and their layout more scattered, which increases classification difficulty. In tropical regions such as Hainan, cloud cover during the rainy season often interferes with image quality. Although a median compositing strategy was applied, spectral confusion still occurred in some areas, reducing classification consistency. As a result, provinces like Hainan and Liaoning showed noticeable fluctuations in Kappa coefficients across different years.

**Table 4:** Accuracy statistics of provincial-level regions in the Chinese mainland.

Region	2010		2016		2022	
	OA	Kappa	OA	Kappa	OA	Kappa
Anhui	0.99	0.78	0.98	0.84	0.98	0.80
Beijing	0.99	0.78	0.97	0.81	0.98	0.79
Fujian	0.99	0.74	0.98	0.74	0.96	0.76
Gansu	0.99	0.80	0.98	0.84	0.98	0.88
Guangdong	0.99	0.79	0.97	0.74	0.98	0.79
Guangxi	0.95	0.80	0.99	0.90	0.99	0.81
Guizhou	0.98	0.85	0.98	0.75	0.99	0.73
Hainan	0.99	0.97	0.97	0.83	0.98	0.87
Hebei	0.96	0.74	0.98	0.90	0.97	0.83
Henan	0.98	0.76	0.98	0.83	0.99	0.81
Heilongjiang	0.98	0.78	0.99	0.91	0.98	0.86
Hubei	0.99	0.83	0.97	0.79	0.98	0.81
Hunan	0.98	0.86	0.98	0.81	0.99	0.84
Jilin	0.98	0.84	0.98	0.91	0.98	0.84
Jiangsu	0.97	0.80	0.97	0.75	0.98	0.85
Jiangxi	0.99	0.76	0.98	0.83	0.98	0.85
Liaoning	0.96	0.79	0.98	0.91	0.94	0.81
Inner Mongolia	0.98	0.75	0.98	0.84	0.98	0.76
Ningxia	0.98	0.80	0.99	0.94	0.99	0.92
Qinghai	1.00	0.77	0.99	0.94	0.96	0.78
Shandong	0.97	0.79	0.97	0.90	0.96	0.86
Shanxi	0.98	0.81	0.98	0.85	0.98	0.82
Shaanxi	0.98	0.78	0.98	0.84	0.97	0.83
Shanghai	0.99	0.74	0.97	0.74	0.95	0.79
Sichuan	0.99	0.84	0.97	0.80	0.95	0.76
Taiwan	0.98	0.75	0.99	0.77	0.99	0.78
Tianjin	0.99	0.89	0.97	0.81	0.97	0.74
Xinjiang	0.99	0.88	0.98	0.90	0.98	0.79
Yunnan	0.99	0.86	0.99	0.96	0.99	0.93
Zhejiang	0.97	0.76	0.97	0.81	0.97	0.81
**Total**	**0.98**	**0.79**	**0.98**	**0.86**	**0.97**	**0.82**

OA, Overall accuracy.

#### Local image contrast

**Fig. 13:**
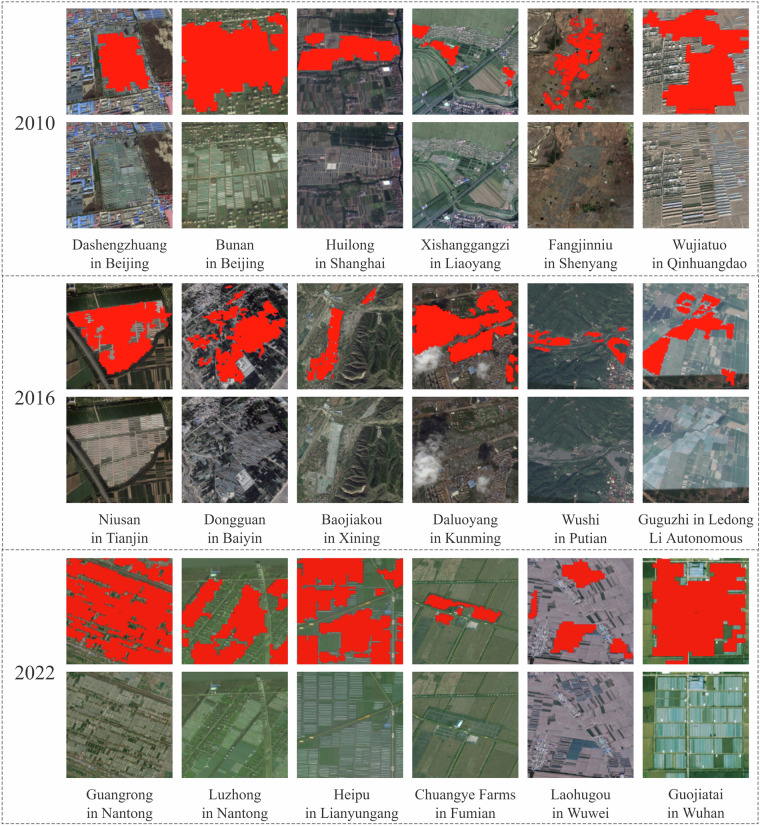
Partial comparison of the classification results of three annual agricultural greenhouse categories. The red part is the classification result of this study.

This study listed the greenhouse classification results of local areas by year and compared them with the original Google Earth remote sensing images of their locations to demonstrate the classification effect of the research dataset more intuitively (Fig. 13). This comparative analysis clearly indicated that the classification results were highly consistent with the actual geographical conditions. This further validated the theoretical effectiveness of the classification technology proposed in this study, indicating its reliability in practical applications.

### Consistency evaluation and accuracy comparison with the existing GH dataset

This study used a random forest model to identify and extract agricultural greenhouses, achieving high overall accuracy. Compared with other studies, our results show improved performance. Feng *et al*.^29^ reported an average classification accuracy of 87.45% for agricultural plastic greenhouses in China in 2019. Huang *et al*.^11^ achieved 92.01% accuracy in Shouguang, Shandong. Wu *et al*.^36^ reported 85% accuracy for suburban greenhouses in Xiaoshan District, Hangzhou. In contrast, our study achieved an average classification accuracy of 98%, which is 10.55%, 5.91%, and 13% higher than the above results, respectively.

**Fig. 14:**
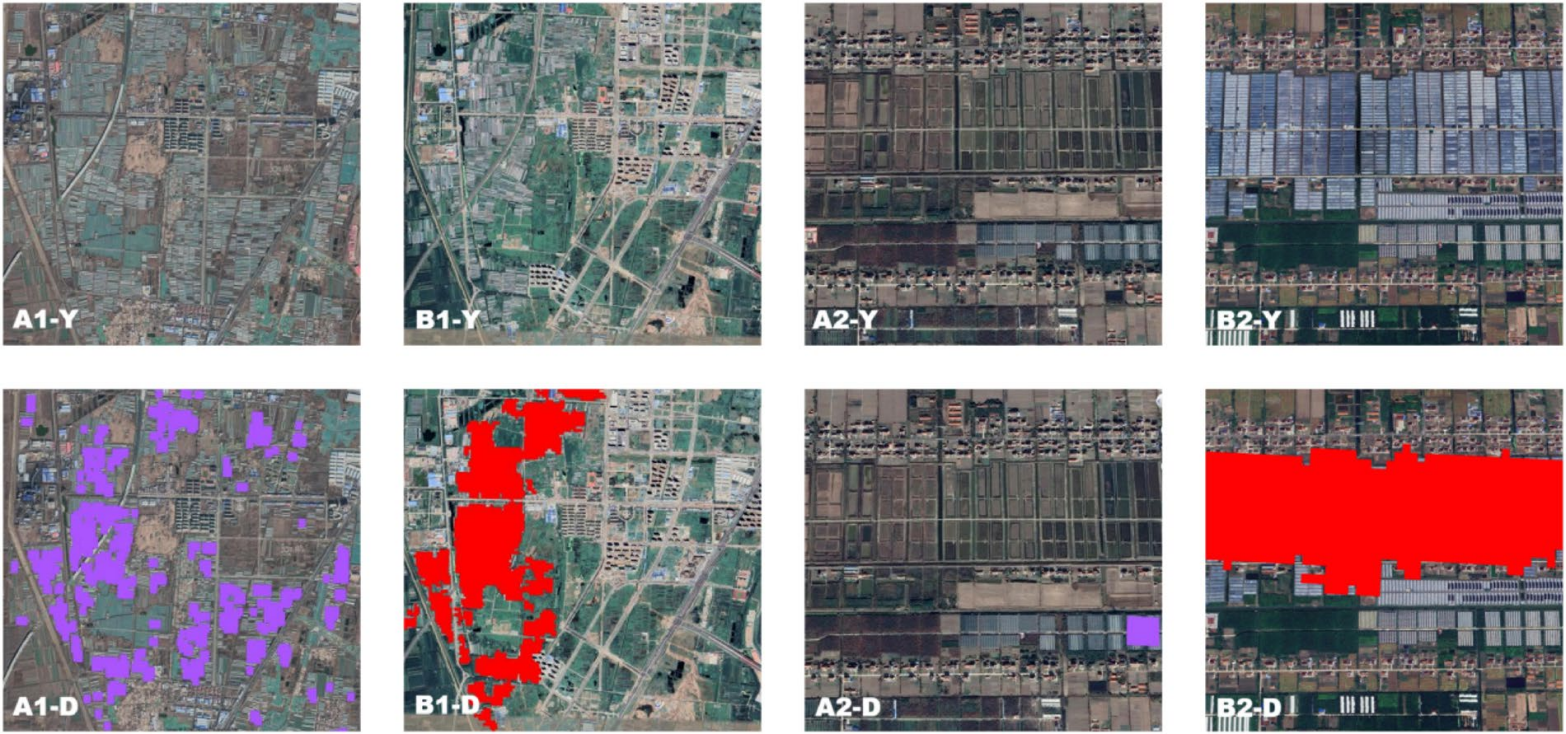
Partial comparison of classification results for the years 2019 and 2022. Note: A represents the year 2019, B represents the year 2022, Y represents the original image, D represents the image after overlaying the greenhouse results, purple represents the greenhouse vector produced by Feng *et al*.^29^ in 2019, and red represents the greenhouse vector produced in this study for the year 2022.

First, the classification results of this study within the same small area were compared with the national greenhouse grid dataset created by Feng *et al*.^29^ (Fig. 13). As shown in Fig. 14, the classification dataset of this study was basically consistent with that of Feng *et al*.^29^, both showing the areas of attenuation and basic maintenance. In 2022, this research dataset accurately corresponded to the areas of newly added greenhouses, reflecting the actual changes in greenhouse space.

Furthermore, this study selected some representative provinces to compare the greenhouse area data in 2016 and 2022 with those in the dataset constructed by Feng *et al*.^29^ in 2019. Overall, the area of agricultural greenhouses still showed a trend of year-on-year growth, which, to a certain extent, also verified the accuracy of the results of this study (Table 5).

**Table 5:** Comparison of greenhouse area in different datasets in typical provinces.

Region	Greenhouse area in 2016 (ha)	Greenhouse area in 2019 (ha)	Greenhouse area in 2022 (ha)
Beijing	14,839.27	12,530	19,371.74
Hebei	57,018.43	41,180	74,808.6
Heilongjiang	56,657	57,860	60,194.68
Jiangsu	12,686.78	46,130	54,696.67
Liaoning	96,129.84	124,700	175,229.97
Inner Mongolia	14,424.78	20,780	21,658.36
Shandong	267,550.42	230,970	281,734.96
Zhejiang	41,329.77	46,370	48,895.25
**Total**	**836,634.94**	**1,032,980**	**1,296,026.79**

Compared with previous studies, the national agricultural greenhouse area in 2022 was 1,295,479.91 ha, which is similar to the 1,262,400.0 ha reported by Wang *et al*.^37^ for 2018. The greenhouse areas in Shandong (290,700.0 ha) and Liaoning (180,100.0 ha) are also very close to the areas extracted in this study, 281,734.96 ha and 175,229.97 ha, respectively. This is slightly higher than the 1,032,980.0 ha reported by Feng *et al*.^29^ for the 2019 national agricultural greenhouse dataset. When compared to agricultural census data, the greenhouse area extracted in this study for 2010 was 555,459.78 ha, which is slightly higher than the 465,086.0 ha from the second agricultural census (2006). The national greenhouse area in 2022 was 1,295,479.91 ha, which is greater than the 981,000.0 ha reported in the third agricultural census (2016). Additionally, the agricultural greenhouse area in Beijing for 2010, extracted in this study (13,308.02 ha), is slightly smaller than the agricultural greenhouse area reported in the 2011 *Beijing Statistical Yearbook*, likely due to the exclusion of some small greenhouses in this study. From a time-series perspective, the differences in the datasets are reasonable, and the classification results have high accuracy.

## Usage Notes

Data application: Data files were imported into Geographic Information System (GIS) software using ArcGIS software. This dataset can be applied to multiple fields: (1) Agricultural planning: The dataset can provide information on the distribution of agricultural greenhouses to government agencies and agricultural entrepreneurs, assisting in agricultural layout and land use planning. (2) Production management: The dataset can assist agricultural producers in understanding the distribution and scale of greenhouses to optimize crop planting and management. (3) Scientific research: Research on the environmental impact of agricultural greenhouses and crop growth conditions can use the dataset in conjunction with other agricultural-related data. (4) Policy-making: This dataset can provide data support for the formulation of agricultural policies, including but not limited to subsidy policies and technical support. Besides these, the data also has application value in disaster assessment. For example, after a natural disaster, the damaged areas of agricultural greenhouses can be assessed to provide data support for postdisaster reconstruction and recovery. In terms of market analysis, the data can help analyze the supply and demand situation of agricultural products, guiding agricultural market pricing and sales strategies. In summary, this dataset can provide solid data support for the sustainable development of agriculture.

## Data Availability

Users can access and obtain GH classification codes by https://github.com/China-GH/ChinaGH, which are used for remote sensing image classification and analysis. At the same time, we also provide a rich dataset of samples for training and testing in GEE. Users can access these basic data and codes, and use professional GIS software such as ArcGIS 10.2, ArcGIS Pro, and so forth, for more in-depth processing and analysis work.
